# Observing the Working Alliance in Videoconferencing Psychotherapy for Alcohol Addiction: Reliability and Validity of the Working Alliance Inventory Short Revised Observer

**DOI:** 10.3389/fpsyg.2021.647814

**Published:** 2021-08-31

**Authors:** Nathálya Soares Ribeiro, Fernando Antonio Basile Colugnati, Nikolaos Kazantzis, Laisa Marcorela Andreoli Sartes

**Affiliations:** ^1^Human Sciences Institute, Psychology Department, Center for Research, Intervention, and Evaluation for Alcohol and Drugs, Federal University of Juiz de Fora, Juiz de Fora, Brazil; ^2^Cognitive Behavior Therapy Research Unit, Institute for Social Neuroscience, Melbourne, VIC, Australia; ^3^Beck Institute for Cognitive Behavior Therapy and Research, Philadelphia, PA, United States

**Keywords:** working alliance, cognitive behavioral therapy, alcohol, COVID−19, telepsychotherapy, eHealth, evaluation, psychotherapeutic process

## Abstract

The coronavirus disease-2019 (COVID-19) pandemic has affected the mental health and alcohol consumption of individuals. Videoconferencing psychotherapy has become a fundamental mode of treatment for people with alcohol use disorders. However, there are still doubts about its effectiveness and the therapeutic relationship. The working alliance is considered a foundation of effective practice in cognitive behavioral therapy (CBT). Observer measurements of the working alliance have demonstrated reliability and meaningful associations with the reduction of symptoms. However, translations of instruments to evaluate the working alliance and examine its construct have not previously been conducted for online psychotherapy for alcohol addiction. This study aimed for the cross-cultural adaptation of the Working Alliance Inventory-Short Form-Observer (WAI-SR-O) for Brazil and the evaluation of its reliability and evidence of its validity in videoconferencing psychotherapy for alcoholism. The WAI-SR-O was applied by pairs of observers for the evaluation of the working alliance in 19 recorded sessions of videoconferencing psychotherapy of 10 clients with a diagnosis of alcohol addiction. The sessions were also evaluated by the therapist (WAI-T) and client (WAI-C). The WAI-SR-O shows a moderate inter-rater intraclass correlation coefficient (ICC = 0.67) for the general scale, higher ICC for the goals and bond subscales, and a moderate value for the task subscale. The internal consistency was good (a = 0.86). The results show low but significant correlations among the goals and bond subscales of the WAI-SR-O and the general, goals, and bond scales of the WAI-T. No correlations were found with the WAI-C. As the literature points out, the client, therapist, and observer versions of the WAI evaluated the alliance differently, requiring further study. The WAI-SR-O proved to be a reliable and valid measurement for the evaluation of the working alliance in videoconferencing psychotherapy for alcohol addiction, becoming an important tool for the study of the working alliance in telepsychotherapy.

## Introduction

The coronavirus disease-2019 (COVID-19) pandemic has brought psychosocial issues that culminated in critical challenges for research, practice, and mental health policy. In the face of this highly damaging context for mental health, especially for those who have already suffered from some disorder, therapists have been forced to migrate to telepsychotherapy to continue psychological treatments. Therefore, telepsychotherapy, that is, the practice of psychotherapy through technologies, is an opportunity to surmount these challenges, enabling access to mental healthcare in such a difficult time (Inchausti et al., 2020; Simpson et al., 2020; Watts et al., 2020). Even before the pandemic, telepsychotherapy has already gained notoriety for allowing practitioners to confront the perceived stigma, and to respond to the insufficient supply of specialized treatments (Clement et al., 2015; Norwood et al., 2018). In substance use disorder (SUD), telepsychotherapy plays an essential role as a modality that reduces the disparities in mental health regarding access to treatment, and is an alternative treatment to cope with stigma, which often hampers the search for treatment (Connery et al., 2020). However, some psychotherapists fear that it is not possible to establish a working alliance in this modality of treatment. On the one hand, this fear is being overcome by the context of the pandemic. On the other hand, some psychotherapists still believe that it is inconceivable to develop an optimum working alliance by means of telepsychotherapy, even when studies show the opposite (Sucala et al., 2013; Berger, 2017; Roesler, 2017; Topooco et al., 2017; Norwood et al., 2018; Connolly et al., 2020; Doorn et al., 2020; Watts et al., 2020). It is noteworthy that studies indicated similar results between online and face-to-face psychotherapies (Andersson, 2016; Carlbring et al., 2018; Karyotaki et al., 2018; Bouchard et al., 2020), and that some participants express a preference to develop an online working alliance rather than a face-to-face one (Watts et al., 2020).

Videoconferencing psychotherapy is the closest to the conventional psychotherapy model among the possibilities (e.g., telephone, chat, e-mails), being one of the methods indicated to overcome barriers of physical distance (Norwood et al., 2018; Watts et al., 2020). It allows audio and video information to be shared simultaneously, enabling the observation of non-verbal behavior and the development of a social presence that promotes familiarity, connectedness, and comfort (Backhaus et al., 2012; Cavanagh and Millings, 2013; Nelson and Duncan, 2015). Reviews indicated that the quality of the working alliance in videoconferencing psychotherapy was similar to that of face-to-face interventions (Backhaus et al., 2012; Singulane and Sartes, 2017). However, studies show some heterogeneity in outcomes. For example, Norwood et al. (2018) found that the quality of working alliance was slightly lower in videoconferencing psychotherapy than in face-to-face. These results highlighted the need for studies on the working alliance in videoconferencing psychotherapy.

Strong therapeutic relationship is considered a necessary basis, but not a sufficient one, for the effective provision of cognitive behavioral therapy (CBT) (Beck, 2011; Kazantzis et al., 2017). The working alliance (also referred to as the therapeutic alliance) is a core element of that therapeutic relationship. This may be defined as the extent to which the client and therapist agree on therapy goals, the tasks to achieve those goals, and relate to one another in a mutually respectful, warm, and encouraging manner (Bordin, 1979). The alliance differs from other therapeutic relationship elements in CBT, such as collaboration, which refers to active client-therapist teamwork. It could be facilitated by therapist behaviors of soliciting client input, shared decision-making, responsiveness to the contributions of the client, and feedback (Dattillio and Hanna, 2012; Okamoto et al., 2019). Therefore, the alliance is a concept that pertains to the agreement on the focus of therapy and a therapeutic bond. In CBT for addictions, clients are generally aware of the social stigma and potential judgment on their substance use or dependence. Therefore, a working alliance is considered especially important for adherence to treatment, motivation readiness, self-efficacy, and consumption reduction (Horvath and Symonds, 1991; Connors et al., 1997; Ilgen et al., 2006; Meier and Donmall, 2006; Kan et al., 2014; Maisto et al., 2015).

The working alliance has been widely studied in online CBT. Alfonsson et al. (2016) showed that good alliance predicts improved anxiety symptoms in online treatment. Anderson et al. (2012) found no differences in the alliance between online and face-to-face CBT for the treatment of anxiety. Moreover, a higher alliance was related to a decrease in anxiety symptoms under both conditions. Reviews point out that it is possible to develop a higher alliance in online CBT, similar to that of face-to-face modality (Sucala et al., 2012; Singulane and Sartes, 2017). King et al. (2014) found no significant differences between face-to-face and online treatment modalities for substance use, neither in the assessment of the therapist nor of the patient. Kay-Lambkin et al. (2017) revealed that a higher alliance was related to a decrease in depression symptoms and cannabis use in online CBT. These findings demonstrate that the quality of the working alliance is of great importance in online CBT for SUDs, enabling online psychotherapy to be a good alternative treatment for this population.

The Working Alliance Inventory (WAI: Horvath and Greenberg, 1989), which is based on Bordin's (1979) conception of the alliance, has emerged as one of the most widely used assessments in this field (Hanson et al., 2002; McCabe and Priebe, 2004; Ardito and Rabellino, 2011; Ribeiro et al., 2019). There are client, therapist, and observer versions of the WAI, all widely known and used by psychotherapeutic process researchers (Hanson et al., 2002), particularly in face-to-face psychotherapy. Furthermore, the WAI shows evidence of convergent validity and optimal psychometric properties, with Cronbach's alpha ranging from 0.86 to 0.98 (Horvath and Greenberg, 1989; Tichenor and Hill, 1989; Cecero et al., 2001; Vöhringer et al., 2013; Andrade-González and Fernández-Liria, 2015a,b; Ribeiro et al., 2019). Nonetheless, the versions mentioned have shown different data regarding the mean score and prediction of therapeutic results. The mean scores of the client version are higher than those of the therapist version, a fact also observed in telepsychotherapy (Watts et al., 2020), although the therapist and observer versions have been more related to psychotherapeutic outcomes (Horvath and Symonds, 1991; Cecero et al., 2001; Fenton et al., 2001; Meier and Donmall, 2006; Bickman et al., 2012; Andrade-González and Fernández-Liria, 2015a,b; McLeod et al., 2017). This phenomenon is called the Rashomon Experience (Caskey et al., 1984; Ardito and Rabellino, 2011) and indicates the need for measurements that assess the working alliance more homogeneously.

The observer versions are highlighted as a suitable option for the most reliable measurement of the working alliance. First, the therapist and client tend to differ on what a good working alliance is, since therapists comprehend the concept, and clients do not. Therefore, observers are trained in the concept of working alliance and the correct use of the measurement to minimize misunderstandings (Ardito and Rabellino, 2011). Second, the observer versions show excellent evidence of validity, with Cronbach's alpha ranging from 0.9 to 0.98 (Tichenor and Hill, 1989; Cecero et al., 2001; Shelef and Diamond, 2008; Vöhringer et al., 2013; McLeod et al., 2017; Ribeiro et al., 2019). Third, the observer versions allow several alliance measurements in the same session without interruption (Vöhringer et al., 2013). Finally, Norcross and Lambert (2014) argued that brief observer measurements of the alliance constitute a promising evaluation methodology. In the case of telepsychotherapy, Norwood et al. (2018) and Watts et al. (2020) highlighted the fact that working alliance data obtained exclusively from clients could be a problem, since psychotherapists usually underestimate the quality of the telepsychotherapy relationship. For these reasons, the WAI Short Revised Observer (WAI-SR-O; Kazantzis et al., 2021) was developed, initially for face-to-face psychotherapy.

Watts et al. (2020) indicated the need to extend the research on measurement of the working alliance in telepsychotherapy, since there is a vast transition of therapists to the online modality owing to the pandemic. Penedo et al. (2019) highlighted that, in most cases, the measurements of the working alliance applied in telepsychotherapy are the same ones used in the face-to-face modality. Thus, the following question arises: to what extent is it possible to employ the working alliance construct applied in traditional therapy to online psychotherapy? Furthermore, this author argued that the psychometric properties of measurements developed for a face-to-face context can produce different results when applied in online psychotherapy, requiring a study on how the measurement of the working alliance works in this modality of treatment. There are two primary studies on the measurement of the working alliance developed for telepsychotherapy. Kiluk et al. (2016) and Penedo et al. (2019) developed the WAI-Tech and the WAI-I, respectively, for self-guided interventions. The WAI-Tech is based on the WAI 36-item version, while the WAI-I is based on the WAI-SR 12-item version, and both are designed to be answered by clients. The sentences are modified to refer to the program and not the therapist. However, there are still several studies that investigate the psychometric properties of alliance measurements in the videoconferencing modality of psychotherapy. It must also be taken into account that technology has introduced new variables to psychotherapy processes that may affect the alliance: a delay in the call can create situations in which the client and the therapist may inadvertently be interrupted; the image may become unstable; there is some difficulty maintaining eye contact (Watts et al., 2020).

Little has been discussed about evidence of the validity and reliability of instruments in telepsychotherapy. Systematic reviews that aimed to analyze measurements of the alliance did not find studies focusing on investigating their psychometric properties in online psychotherapy (Elvins and Green, 2008; Ardito and Rabellino, 2011; Ribeiro et al., 2019). From a psychometric standpoint, it is imperative to investigate how a measurement works in different contexts (American Educational Research Association et al., 2014). Hence, it is necessary to develop valid working alliance measurements available for videoconferencing psychotherapy. In Brazil, there are a few working alliance measurements evaluated regarding their psychometric properties (Marcolino and Iacoponi, 2001; Araújo and Lopes, 2015), but none evaluated in the treatment provided via internet. Hence, it is necessary to develop valid working alliance measurements available for videoconferencing psychotherapy. This study aims to develop the cross-cultural adaptation of the WAI-SR-O to Brazilian Portuguese and the evaluation of its reliability and evidence of its validity for alcohol addiction in videoconferencing psychotherapy.

## Materials and Methods

### Sample

The study included 19 video recordings of 10 clients who received brief CBT for alcohol use disorder. The alliance was evaluated at the beginning, middle, and end of treatment (sessions 4, 8, and 12, respectively), considering that the working alliance is developed from the third session on, and that it is a construct that involves ruptures and recoveries during the psychotherapeutic course (Safran and Segal, 1990; Luborsky, 1994; Garfield, 1995; Samstag et al., 2004; Safran and Muran, 2006). If any recording were damaged, it could be replaced by a closed session (with a maximum of one earlier or later interval). Three of the clients dropped out of the treatment, making it impossible to record sessions 8 and 12, and some initial sessions were excluded because of recording problems. Therefore, the recording sample was composed of sessions 3 (5.3%), 4 (42.1%), five (21.1%), 8 (84.2%), 11 (42.1%), and 12 (21.1%). Considering that each session was evaluated by two observers, there are a total of 38 observations. Table 1 shows the sample characteristics.

**Table 1:** Description of clients, therapists, sessions and general scores evaluated by pairs of Working Alliance Inventory-Short Form-Observers (WAI-SR-O), Working Alliance Inventory therapists (WAI-T), and Working Alliance Inventory clients, (WAI-C) and the scores observed of the general scales.

**Client**	**Therapist**	**Initial session**			**Middle session**			**Final session**		
		**General scores**								
		**WAI-SR-O**	**WAI-T**	**WAI-C**	**WAI-SR-O**	**WAI-T**	**WAI-C**	**WAI-SR-O**	**WAI-T**	**WAI-C**
1	A	–	–	–	II−12 VII−16	84	84	II−12 V−13	84	84
2	B	I−12 V−12	72	83	III−18 V−12	70	83	–	–	–
3	A	I−14 V−13	84	83	I−12 V−13	84	81	–	–	–
4	A	III−14 IV−12	80	84	III−15 IV−13	81	83	I−12 IV−12	81	84
5	B	–	–	–	I−14 II−12	73	–	I−12 VII−21	73	–
6	B	II−15 VI−12	76	82	VI−12 VII−18	74	82	V−12 VI−14	74	84
7	C	–	–	–	IV−12 VII−20	–	84	IV−13 VI−16	67	84
8	B	II−12 VII−24	77	75	–	–	–	–	–	–
9	C	V−19 VI−15	56	–	–	–	–	–	–	–
10	A	II−12 VII−20	74	81	–	–	–	–	–	–
M ± SD		14.7 ± 3.7	74.1 ± 8.9	81.3 ± 3.3	14.2 ± 2.8	77.7 ± 6.1	83.0 ± 1.3	13.7 ± 2.9	75.8 ± 6.8	84.0 ± 0

*Therapists: A, B, C. Observers I to VII ordered by time of clinical experience (none to 15 years). Score WAI-SR-O Min 12 Max 60, WAI-T, and WAI-C Min 12 Max 84*.

*N of WAI-SR-O = 38; WAI-T =18; WAI-C = 16*.

The participants came from *Programa Álcool e Saúde*, a project of the Center for Research, Intervention and Evaluation for Alcohol and Drugs (CREPEIA) in the Federal University of Juiz de Fora, in Brazil. The study embraces two clinical trials aiming to evaluate the same protocol based on Project MATCH (Matching Alcoholism Treatment to Client Heterogeneity; Kadden et al., 1995), composed of 12 sessions, in online CBT (Cançado, 2017; Gumier, 2019). This study is registered in the Brazilian Clinical Trials Registry (http://www.ensaiosclinicos.gov.br/rg/RBR-74jtvm/). The study included individuals whose age ranged from 18 to 65 years and with a diagnosis of alcohol use disorder. Individuals who had not used alcohol in the last 30 days, reported a diagnosis of another substance use disorder (except tobacco), had taken part in psychotherapeutic treatment in the previous 3 months, or were diagnosed with severe psychiatric disorders were excluded. The sample, altogether, includes 94.7% of men and the mean age of the participants was 44.2 (*SD* = 8.5). Most of the participants (52.6%) had a high level of education. Regarding computer usage, 52.6% reported using computers almost every day, 42.1% of them without any difficulty. About 84.2% adhered to the treatment, in other words, attended at least eight sessions (Ballegooijen et al., 2014).

Treatment adherence was 70%, considering the clients who filled at least eight sessions. Among the 10 clients, 7 completed the treatment and completed the 12 sessions. At the end of the treatment, 5 of the 10 clients answered a brief questionnaire about treatment satisfaction. The data of the other five clients were missing. Around 60% of the clients evaluated the treatment as very satisfactory and 80% reported being very satisfied with the number of sessions and the therapist. About 60% were very satisfied with treatment results and tasks. None of them reported being dissatisfied with the treatment in any item.

### Therapists

Three therapists took part in this study. All of them have a master's or doctoral degree in Psychology and have a CBT qualification for SUD treatment. They each have at least 2 years of clinical experience. They received training in MATCH protocol, which lasted about a month, and had weekly supervision with a PhD therapist who has 15 years of clinical experience (Cançado, 2017; Gumier, 2019).

### Clinical Observers

Seven clinical observers were selected, four psychologists, and three students from the last semesters of Psychology. They have different levels of clinical experience, from none (Observer I) to 15 years (Observer VII). Three of them were also therapists on the project, which required some methodological precautions. To this end, the sessions were randomized, preventing therapists from evaluating their own sessions. All the observers received training in the use of the WAI-SR-O carried out by the principal researcher of this study.

### Treatment

The Matching Alcoholism Treatment to Client Heterogeneity protocol (Kadden et al., 1995) comprises 12 CBT sessions for alcohol use disorder. The first seven sessions are focused on goal establishment, patient psychoeducation in concepts related to alcoholism, recognition of risk situations, discussion of motivation strategies, training in coping skills and problem solving, self-monitoring tasks of alcohol use, and cognitive processes in these situations. The themes of the following four sessions are defined according to client needs. There are 14 possible themes, which are dealing with negative thoughts, family involvement, risk situations, and anger management. The last session involves a summary of strategies during sessions, and the therapist and patient share feedback (Gumier, 2019). The protocol includes baseline and final evaluation, besides a 3-month follow-up evaluation of socio-demographic data, alcohol use, readiness for change, psychiatric symptoms, and beliefs about the effects of alcohol.

The treatment was provided as in traditional therapy but by means of videoconferencing. The themes mentioned were addressed during the session, in synchronous mode, via Skype or Google Hangouts, according to client preference. The between session intervention was sent by email or WhatsApp message, also according to client preference. The therapist and client made an initial agreement, which includes (a) the client was free to contact the therapist to clarify doubts, or even in an emergency, using these apps or phoning the therapist; (b) the client could reschedule his session in case of unforeseen events, provided it was for the same week. If it was not possible to schedule for the same week, an elective session was deducted for each absence of the client, since it is expected that the treatment happens in exactly 12 weeks; (c) if a client had three absences, he was suspended from treatment, understanding that treatment could be compromised if more sessions were discounted. In this case, the client was referred for another treatment or could return after 6 months. This control was necessary because this study is an arm of a study of effectiveness.

### Instruments

*Socio-demographic questionnaire:* includes client information regarding age, sex, socioeconomic class, and schooling.

*Internet use questionnaire:* the researchers developed two questions about the frequency and degree of difficulty regarding internet use.

*Working Alliance Inventory-Short Form-Observer (Kazantzis et al.*, *2021**):* this was derived from the client version of the WAI-Short Revised (WAI-SR; Hatcher and Gillaspy, 2006), consisting of 12 items about the agreement between therapist and client regarding therapy goals (e.g., *There is an agreement on what is important for the client to work on*) and tasks(e.g., *The client believes that the way they are working with his/her problem is correct*), as well as the presence of a bond (e.g., *The client and the therapist respect each other*) (Bordin, 1979). Each of the subscales has four items. It is evaluated on a five-point Likert scale, and a clinical observer answers the instrument. The overall score varies from 12 to 60; the lower the score, the better the quality of the working alliance.

The Working Alliance Inventory-Short Form-Observer was subjected to cross-cultural adaptation in compliance with Beaton et al. (2000) guidelines. The WAI-SR-O, in the original version, was translated to Brazilian Portuguese by two translators, and these translations were synthesized and submitted to back-translation. The resulting version was discussed among the researchers, the author of the original version of the WAI-SR-O, and the back-translator. Afterward, it was evaluated by an expert committee composed of PhDs. Psychologists with clinical experience, guided by a semi-structured questionnaire composed of questions about the WAI-SR-O guidelines and items.

*Working Alliance Inventory-Therapist and WAI-Client (WAI-T and WAI-C; Horvath and Greenberg*, *1989**):* It was applied the Portuguese versions of WAI-C and WAI-T translated by Paulo Machado and Cristiano Nabuco de Abreu. These instruments have 36 items, which must be answered on a seven-point Likert scale. The items are divided into goals, tasks, and bond subscales, each of them with 12 items. Some examples of WAI's items are “*I am worried about the outcome of these sessions*” (goals subscale of the version for the client), “*My client and I both feel confident about the usefulness of our current activity in therapy*” (task subscale of the version for the therapist), and “*I appreciate (the name of the client) as a person*” (bond subscale version for the therapist). They are self-administered by the therapist and the client. The overall score varies from 36 to 252. The convergent analysis of this study only involved the scores of 12 items of the WAI-C and WAI-T, corresponding to the WAI-SR-O items. Thus, the WAI-C and WAI-T general score varied from 12 to 84 and the subscale score from 4 to 28. The higher the score, the better the quality of the working alliance.

*Satisfaction assessment:* Seven questions were asked to assess the satisfaction of the individual with the intervention and the therapist at the end of the intervention.

### Procedures

Clients signed the Term of Consent, written in compliance with the Research Ethics Committee of the Federal University of Juiz de Fora (protocol number 1.360.973).

**Figure 1:**
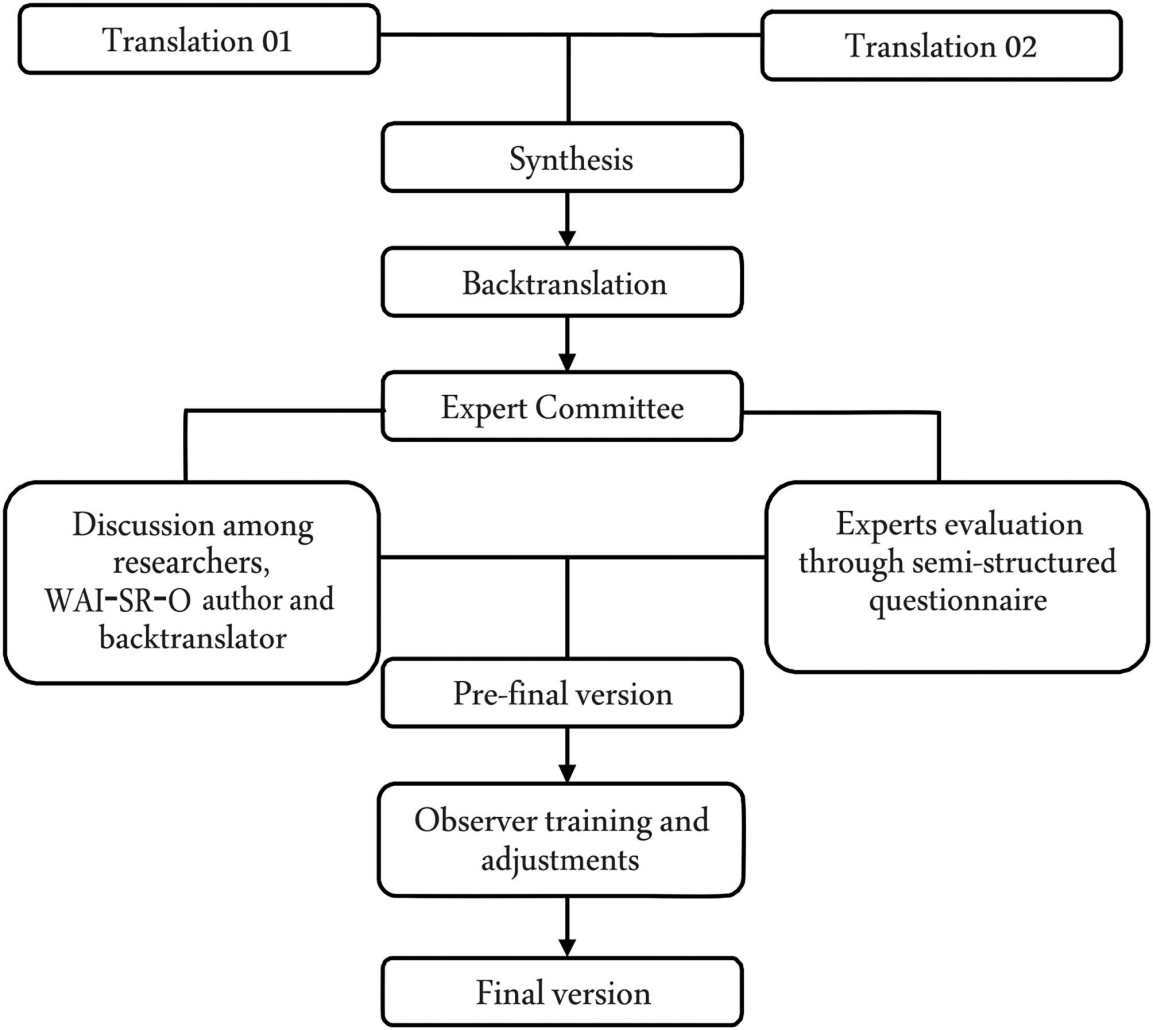
Methodological procedures of the Working Alliance Inventory Short Revised Observer (WAI-SR-O) cross-cultural adaptation.

First, training was given to the seven clinical observers. The training was conducted by the principal researcher of this study. The Brazilian version of WAI-SR-O was used in the training of the observers. The training lasted 8 h and was divided between a theoretical presentation about the working alliance concept and the application of the WAI-SR-O. In this training, the WAI-SR-O was applied in the first 15 min of the sixth session with the client, treated by different therapists, in accordance with the study by Kazantzis et al. (2021). Divergences on the evaluation were discussed among the observers, including the principal researcher, and adjustments were made to the WAI-SR-O, resulting in its final version. This version was subjected to analysis of evidence of validity. The cross-cultural adaptation of WAI-SR-O is summarized in Figure 1.

For the WAI-SR-O evidence of validity study, the entire sessions were watched. The recordings were distributed by non-intentional convenience sampling among the seven evaluators, considering that some of them were also therapists on *Programa Álcool e Saúde* and had their sessions included in this study, as explained previously. Of note is that a researcher carried out this distribution blinded. The WAI-SR-O evaluation occurred in pairs, randomly selected.

### Statistical Analysis

The data were transferred to the 22nd version of Statistical Package for the Social Sciences (SPSS) through double data entry. Descriptive analyses and convergent analysis were carried out among the WAI-SR-O, WAI-T and WAI-C, as well as the coefficient of internal consistency. The 14th version of Stata was used for the intraclass correlation coefficient (ICC) and mean rating analysis.

Generalized linear mixed models (GLMMs) with multilevel structure were used to evaluate the inter-rater reliability/precision (McCulloch and Searle, 2001) of the WAI-SR-O, given the unbalanced design provided by the randomization of observers for each session, an assumption for classical ICC estimates. The model was adjusted for observers and therapist as fixed effects, and random effects for observers nested in dyads (client + therapist). The fixed effect for each client session (4th, 8th, and 12th) was tested, but it was removed from the analyses because it was neither clinically relevant nor statistically significant. The number of sessions evaluated per client was quite different at the end of the study, given the rate of attrition in the follow-up. Therefore, we only calculated the inter-rater reliability and not the intra rater reliability. The ICC ranges from 0 to 1, and the closer to 1, the better the reproducibility (inter), and is interpreted in accordance with Shrout and Fleiss (1979).

Since the instruments used for evidence of validity with external variables did not have validation studies for Brazil, we calculated the intra rater ICC of each general, goals, task, and bond scale of the WAI-T, and of the general scale of the WAI-C, measuring the agreement between the 4th, 8th, and 12th sessions for the same individual. We also used the GLMM for assessment of the ICC, however, with randomized effects of the dyads. In client evaluations (WAI-C), it was not possible to use any model to calculate the other ICCs, since the evaluation variability was excessively small. Spearman correlations were also used among WAI-SR-O, WAI-T, and WAI-C scores, and the mean of the general and subscale scores was calculated.

## Results

### Reliability/Precision and Internal Consistency

**Figure 2:**
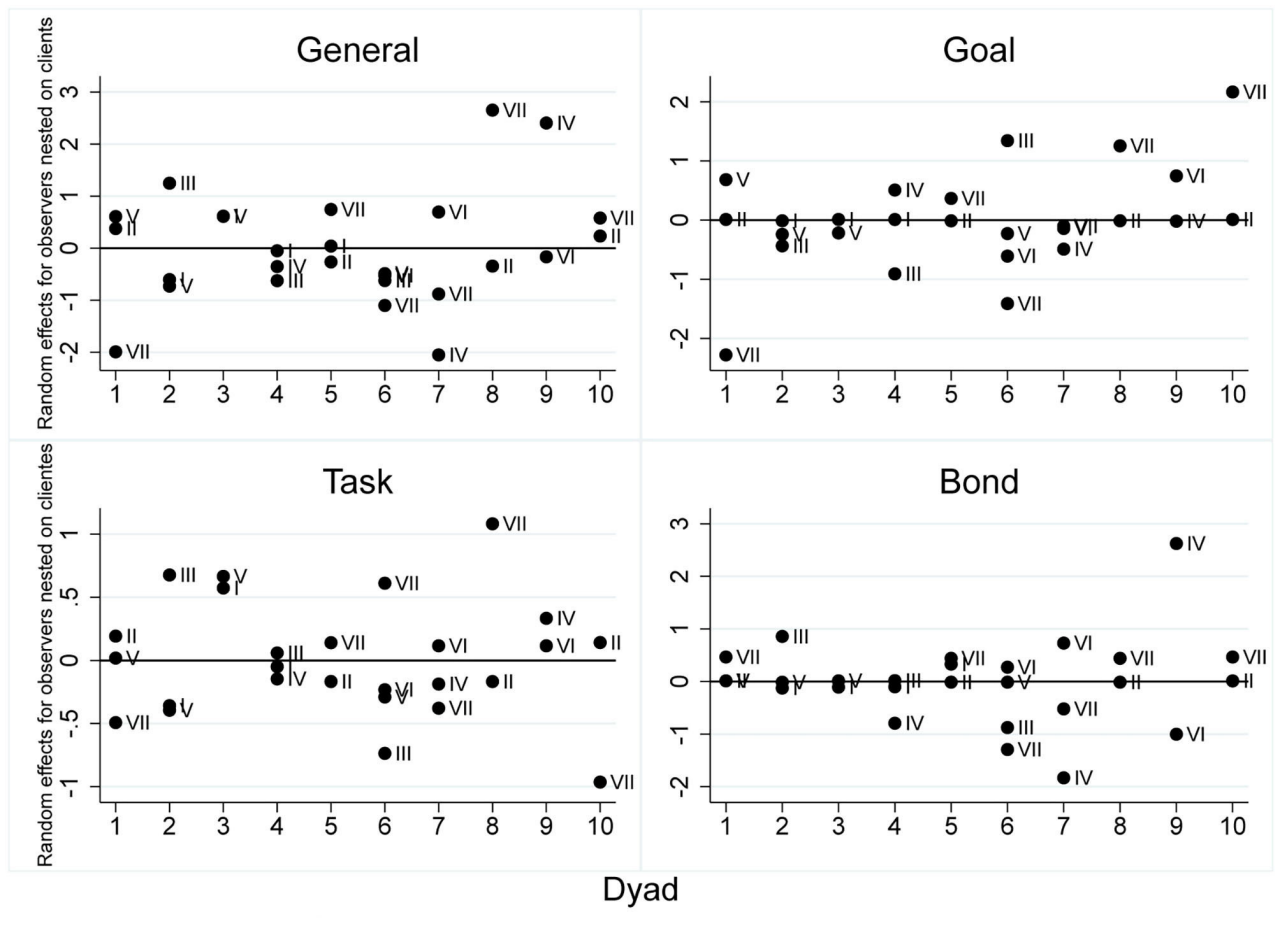
Weighted averages of the WAI-SR-O for the general scales and subscales. The study embraces two clinical trials aiming to evaluate the same protocol. 1–10: clients; I–VII: observes.

The Working Alliance Inventory Short Revised Observer inter-rater intraclass correlation coefficient was calculated to evaluate the reproducibility. The ICC of the objective subscale was 0.89 (95% CI 0.72–0.96), and that of the bond subscale was 0.87 (95% CI 0.67–0.95), indicating a particularly good agreement in these areas. However, in the task subscale, the ICC was 0.47 (95% CI 0.06–0.93), which impacted the ICC of the general score (0.67, 95% CI 0.31–0.91), regarded as substantial (Shrout and Fleiss, 1979). Figure 2 shows the random effects of each observer. There are 10 client/therapist dyads on the x-axis, and on the y-axis there is the scale of the random effects, with a standardized mean of zero. It should be noted that the dyads of observers varied according to the session evaluated for each client. For that reason, some clients have more than two observers, as described in Figure 2, considering all the observations done throughout the psychotherapeutic process.

The inter evaluator adjusted means of the WAI-SR-O general scores had a significant variability, with a minimum value of 12.3 (*SD* =0.79) and a maximum of 19.78 (*SD* =0.65). A similar difference was verified with the subscales [Goals: *M* = 3.73 *SD* = 0.61 (min.), *M* = 6.82 *SD* = 0.38 (max.); Tasks: *M* = 3.94 *SD* = 0.44 (min.), *M* = 7.28 SD = 0.37 (max.); Bond: *M* = 4.18 *SD* = 0.46 (min.), *M* = 5.65 *SD* =0.37 (max.)]. The data are shown in Table 2.

**Table 2:** Adjusted mean, intraclass correlation coefficient, and confidence interval of the WAI-SR-O, WAI-T, and WAI-C measurements.

**Scales**	**Adjusted mean ± SE**	**ICC**	**Confidence interval (95%)**
**WAI-SR-O**	**Inter rater**		
General score	12.9 ± 0.84	0.67	0.31–0.91
Goals score	4.0 ± 0.50	0.89	0.72–0.96
Task score	4.8 ± 0.45	0.47	0.06–0.93
Bond score	4.1 ± 0.48	0.87	0.67–0.95
**WAI-T**	**Intra rater**		
General score	74.2 ± 2.5	0.99	0.98–0.997
Goals score	24.1 ± 1.1	0.98	0.94–0.99
Task score	23.4 ± 1.1	0.996	0.991–0.998
Bond score	26.7 ± 0.4	0.94	0.84–0.98
**WAI-C**			
General score	82.0 ± 0.97	0.94	0.82–0.98

Table 1 gives the general scale scores per client and session. In the WAI-SR-O there was a divergence between the scores generated by some pairs of observers, especially when observers 3 and 7 were included. These observers had identified more problems in the working alliance during the session. Observer 7 was the most clinically experienced, and observer 3 had less clinical experience but greater knowledge of the working alliance. However, there is a greater balance among the other observers. Figure 2 shows the random effects, which could be understood with standard deviations about a general mean adjusted by the model of WAI-SR-O, higher divergence in the task subscale, and higher convergence in the subscales of goals and bond. This divergence explains the lower relative agreement (0.47) found on the general scale of the WAI-SR-O, and indicates problems with calibration and the need for further training in the use of the WAI-SR-O.

Regarding therapist evaluation through the WAI-T, high values were found for the alliance and little variation from one session to another. In contrast, the WAI-C evaluations were particularly high and constant. The agreement between the assessments made by the therapists on the 4th, 8th, and 12th sessions combined, for the same client, was 0.99 (SE 0.003, 95% CI 0.98–0.99) for the general scale, 0.98 (SE 0.01, 95% CI 0.94–0.99) for the goal subscale,0.99 (SE 0.001, 95% CI 0.991–0.998) for the task subscale, and 0.94 (SE 0.03, 95% CI 0.84–0.98) for the bond subscale. All the ICCs were rated high on the WAI-T. The intra rater ICC for the general score of the WAI-C was high (0.94, SE 0.34, 95% CI 0.82–0.98). The data relating to the ICC are shown in Table 2. Internal consistency analysis of the 12 items of the adapted WAI-SR-O indicated an adequate Cronbach's alpha (α = 0.86).

### Relationship Among Observer, Therapist, and Client Measurements

Considering the good repeatability of the WAI-T and WAI-C (general scale), we conducted correlation analysis to analyze convergent validity. For the correlation analysis, it was decided to change the signs of the WAI-SR-O in order to clarify the results. The general scale of the WAI-SR-O showed higher correlations with its three subscales. The subscales showed moderate correlations among themselves. The goals and bond subscales of the WAI-SR-O showed weak but significant correlations with the scales of the WAI-T, except for the bond subscale. There was no correlation between the WAI-C and the WAI-SR-O. The analysis found moderate to high correlations among the general scale and the subscales of the WAI-C, and between the goals and task subscales. The goals subscale of both the WAI-T and the WAI-C had a limited but significant correlation. The results are shown in Table 3.

**Table 3:** Spearman correlations of WAI-SR-O, WAI-T, and WAI-C.

**WAI**	**Spearman correlations**												**M ± SD**
	**1**	**2**	**3**	**4**	**5**	**6**	**7**	**8**	**9**	**10**	**11**	**12**	
1.WAI-SR-O General													15.10 ± 4.93
2.WAI-SR-O Goals	0.80*												5.25 ± 2.44
3.WAI-SR-O Task	0.80*	0.38*											5.13 ± 1.99
4.WAI-SR-O Bond	0.79*	0.55*	0.66*										4.73 ± 1.16
5.WAI-C General	0.07	0.10	0.15	0.13									81.81 ± 3.24
6. WAI-C Goals	0.10	0.01	0.24	0.01	0.71*								27.89 ± 0.40
7. WAI-C Task	0.10	0.09	0.18	0.09	0.71*	0.68*							27.76 ± 0.53
8. WAI-C Bond	0.02	0.20	0.04	0.06	0.74*	0.25	0.21						26.48 ± 2.76
9.WAI-T General	0.18	0.36*	0.11	0.25*	0.03	0.28	0.22	0.35					74.61 ± 7.01
10.WAI-T Goals	0.20	0.35*	0.09	0.24*	0.09	0.35*	0.28	0.31	0.99*				24.35 ± 3.06
11.WAI-T Task	0.19	0.41*	0.14	0.26*	0.09	0.16	0.09	0.43*	0.96*	0.92*			23.57 ± 3.21
12.WAI-T Bond	0.17	0.28	0.002	0.20	0.13	0.19	0.21	0.52*	0.87*	0.88*	0.79*		26.70 ± 1.09

* *p < 0.05*.

## Discussion

This study evaluated the reliability of the WAI-SR-O in its Brazilian version, and the internal consistency and relationship with therapist and client measurements. The results are innovative given that the instrument was applied to assess the working alliance in videoconferencing psychotherapy for alcohol dependence. The results show that the goals and bond subscales had a higher agreement among the observers, and that the WAI-SR-O had a higher internal consistency regarding Cronbach's alpha. The internal consistency was maintained a higher correlation among the WAI-SR-O subscales and general scale. However, we only found a moderate agreement among the observers in the task subscale, and the relationship with the external variables shows that the WAI-SR-O had a weak correlation with the therapist version and did not correlate with the client assessment.

This study represents an effort to clarify some aspects of the measurement of the working alliance by the internet, but it is essential to emphasize that the results presented here must be considered in light of their limitations. The small sample hinders the generalization of the results, which must be considered with caution. Furthermore, it is known that the measurements used as external criteria should demonstrate strong validity and reliability evidence, representing a standard gold measurement. Nonetheless, in Brazil, we do not have any measurements that fulfill that criterion. Hence, we opted to use the therapist and client version of WAI, as it is the instrument primarily used in the world, represents the same construct of WAI-SR-O, and is already translated into Portuguese. Finally, the fact that the therapists are also the observers is noteworthy. Although the therapists did not evaluate their own sessions, we acknowledge that being a therapist and observer can insert a bias that cannot be evaluated in this study, since they are considered in the same parameter as the other observers who were not therapists. For this reason, as described in the statistical analysis, observers' random effects estimate the ICCs are nested on dyad/session, which makes them independents given the dyad/session. This fact implies that ICC estimates should be as less biased as possible regarding this observer-therapist dependence, which never happened by design for a given dyad/section. Also, the model included fixed effects for therapists and observers to control for a likely residual bias, or possibly reduce it.

The results regarding the analysis of inter-rater agreement of the WAI-SR-O for videoconferencing psychotherapy indicated good reproducibility of the instrument, but attention should be given to the task subscale, which reached lower values. Studies point out that the items for goals and tasks are confounded, which corroborates the factorial issues in WAI version (Ardito and Rabellino, 2011; Ribeiro et al., 2019). During the observer training, the main doubts of the observers were about the task items. Often, they swapped the task items with goals items. We discussed this fact widely, but it was insufficient to reach better agreement values in this subscale. Osborne (2010) found similar results during the development study on the WAI-SR-O, evaluated for face-to-face psychotherapy. Osborne (2010) aimed to test the training of clinical observers of the WAI-SR-O to calibrate the instrument. Three recalibrations were developed with eight evaluators with different levels of clinical experience. Values found were moderate to high in the first 10 evaluations, decreased in the second recalibration with a further 10 sessions, and obtained moderate to high values in the third recalibration, totaling 30 sessions evaluated. The final ICC shows high values for the general and subscale scores and the task subscale (ICC = 0.54 to 0.74). As in the Osborne (2010) results, the task subscale had a lower ICC in the Brazilian version of the WAI-SR-O. For this reason, the recalibration of the Brazilian WAI-SR-O is relevant in future studies. However, Hatcher and Gillaspy (2006) pointed out that the task subscale had differentiated the task dimension of the working alliance construct better. Thus, the factor structure of the WAI-SR-O should be investigated in order to clarify this issue.

There are many factors that may be involved in the variability of ICC results of the WAI-SR-O in its Brazilian version: (a) better training with evaluators could be necessary, especially for the task subscale; (b) as carried out in Osborne (2010), further recalibrations should be proposed until a suitable level of agreement among evaluators is found; (c) the quality of the recordings may have affected the understanding of some parts of the video; (d) the difference in clinical experience among evaluators, which varied from graduate to postgraduate students and a supervisor, may have affected the required level as to what a good alliance is. It was possible to observe two outliers in the results. One of the observers had more clinical experience and the other had more knowledge of the working alliance construct but less clinical experience, indicating that these characteristics can affect the evaluation of the alliance and must be considered; (e) previous studies showed problems regarding the factorial structure of other versions of the WAI (observers, client, therapists) (Hatcher and Barends, 1996; Corbella and Botella, 2004; Burkard et al., 2009; Munder et al., 2009; Soygüt and Uluç, 2009; Corbella et al., 2011; Falkenström et al., 2014, 2015; Mallinckrodt and Tekie, 2015; Miragall et al., 2015; Smits et al., 2015; Fuertes et al., 2017; Ribeiro et al., 2019), which could make it difficult for observers to recognize several elements corresponding to the goals, tasks, and bond dimensions.

The analysis of inter evaluator reliability demonstrates the extent of the consensus on using an instrument by those administering it, since human judgments are subject to errors of measurement, which can significantly affect the interpretations of the findings (Shrout and Fleiss, 1979). Osborne (2010) argued that the training guidelines of the instrument may affect its usefulness. Therefore, it is fundamental that the WAI-SR-O application training be restructured, making further calibrations to better investigate inter evaluator consistency. In this way, the peculiarities of the working alliance in online CBT should be clarified to improve its assessment in this modality of treatment. This is only possible with more studies on working alliance instruments in telepsychotherapy. Regarding the assessments of the client and the therapist, the intra rater ICCs of the WAI-C and WAI-T were high, indicating good repeatability of these instruments during the therapy;, therefore, we performed correlation analyses with the WAI-SR-O. These results are probably because the evaluator is the same during the psychotherapeutic process. The evaluations of the alliance did not seem to have been affected by the fact that the treatment was carried out via the Internet and the dynamics of the alliance. The therapist evaluation was also consistent, although with a little more variability than the client evaluation. On the other hand, considering that the working alliance is a dynamic construct, varying throughout the sessions, the high stability of the WAI-T and WAI-C assessments was not expected. The low correlations among the WAI-T, WAI-C, and WAI-SR-O were also unforeseen. When comparing the results of the three measurements, the WAI-SR-O and WAI-T seem to have evaluated the alliance more positively than the WAI-C. The most critical assessment of the alliance was that of observers. Previous studies have shown that clients tend to overestimate the working alliance in both online and in face-to-face psychotherapy, and that therapists tend to be more rigid, underestimating the relationship in telepsychotherapy (Singulane and Sartes, 2017). Furthermore, the correlation among observers, therapists, and clients has no consensus, according to the review of the literature by the authors (Ribeiro et al., 2019). Cecero et al. (2001) and Vöhringer et al. (2013) found significant correlations between the WAI-Observer (WAI-O) and the WAI-T, while Vöhringer et al. (2013) found significant correlations between the WAI-O and the WAI-C. Cecero et al. (2001) did not find any correlation between the WAI-C and any other working alliance measurement. The results shown reaffirms the incongruity between these three evaluation perspectives, requiring further studies in this regard. The results indicate that assessing the working alliance by a suitably trained external observer can be crucial for a better evaluation of the working alliance.

This study indicates that the low variability ratings among client evaluations could be associated with social desirability. Thus, clients always evaluate the relationship as positive or extremely positive, although methodological measurements have avoided this bias. Studies have argued that, unlike the therapist, the client does not understand the working alliance construct, which may lead to a limitation of these evaluations (Ardito and Rabellino, 2011). In addition, the absence of variability in client responses compromised the potential for correlation analysis, which may have affected the result. In any case, the results suggest that, although the WAIs are instruments developed to be theoretically similar, observers, clients, and therapists seem to have evaluated the working alliance in telepsychotherapy in different ways. Regarding the internal consistency of the adapted WAI-SR-O, Cronbach's alpha is close to the value of the original measurement (α = 0.88). In the original study, Finn's *r* was 0.91 (Kazantzis et al., 2021). This result, in conjunction with the good correlations between the WAI-SR-O general scale and subscales, shows evidence of a good internal structure. However, factorial analysis or the item response theory can add more information about the structure of the construct of the WAI-SR-O. For this, a large sample is required. Note that the study of the factorial structure of an observer alliance measurement is rare, especially in the field of telepsychotherapy (Ribeiro et al., 2019).

From a future perspective, a study with a larger sample is needed to consolidate the evidence of validity and reliability of the WAI-SR-O. Furthermore, it is necessary that greater attention should be paid to the training of observers to perform WAI-SR-O recalibrations and find more support for the issues raised regarding the task subscale. Such future research should focus on the comparison of videoconferencing psychotherapy and other psychotherapeutic modalities, with face-to-face to self-guided interventions.

Given the relevance of evaluating the working alliance in videoconferencing psychotherapy, it is important to have tools that contribute to the excellence of service for individuals who suffer from alcohol addiction, aiming to improve the quality of the alliance. That is why the adaptation of the WAI-SR-O is so essential, and it is an innovation of this study. Besides that, through this study, the nuances of the evaluation from the viewpoint of the observer could be analyzed and discussed, bringing new knowledge to this area that seems promising, since it is more and more frequently concluded that the evaluation of the alliance from the viewpoint of the client and the therapist differs substantially. The variability of the sample in terms of sociodemographic and even cultural characteristics, considering that they are from different places in Brazil, contributes to the evidence of the validity of the WAI-SR-O.

This study, albeit in a preliminary way, pointed to positive results of telepsychotherapy for alcohol addiction. All the clients evaluated were satisfied with the videoconferencing psychotherapy. The adherence was higher than that found in most studies on alcohol use disorder treatment, which averages around 50% (Lin et al., 2019). The same rate is found in the literature for patient dropout in face-to-face treatment, but in this study, we have found lower values. This seems to be a good indication of the adherence of people with alcohol use disorder to telepsychotherapy. However, the size of the sample limited the conclusions and generalization of the results. It should be noted that most patients drop out by the fifth session, as addressed in the literature (Tarp et al., 2017). We suggest that the higher adherence and satisfaction rates may be related to a good assessment of the working alliance in online therapy by clients, therapists, and observers.

Finally, looking at a future perspective, the results of this study may contribute to the knowledge of videoconferencing psychotherapy, which has been fundamental during the COVID-19 pandemic, and probably will be after it. The COVID-19 pandemic was an unpleasant surprise that generated damage to physical and mental health that should not be ignored. DiClemente et al. (2020) pointed out an increase in alcohol consumption during this crisis, reinforcing the need to expand the access to treatment for this population through the Internet. However, transition to online service was carried out abruptly and without prior training (Inchausti et al., 2020). As Blumenstyk (2020) stated, this “black swan” moment leads to an essential change in the perspective of mental healthcare, bringing online care more forcibly to the discussion. In other words, videoconferencing psychotherapy and other forms of telepsychotherapy had, until the pandemic, encountered several barriers permeated by myths regarding its insertion in clinical practice. Although it is possible that online therapy will continue to be more widely used, even after the control of or end of the pandemic, few studies provide evidence of the effectiveness and the therapeutic relationship in online CBT with the participation of the therapist (Kay-Lambkin et al., 2011; Postel et al., 2015; Gumier, 2019; Singulane and Sartes, 2021). Therefore, it is essential that questions about this therapeutic modality be clarified. The pandemic gave us no choice but to accept telepsychotherapy, which, more than ever, requires investment in research to better understand the nuances of this modality of treatment and, as a consequence, to prepare psychologists for better utilization of this resource (Simpson et al., 2020). In these terms, we believe that the WAI-SR-O can be a relevant tool for the study on the working alliance in online CBT and also the training of therapists in it.

## Conclusion

Studies on measurement of the working alliance of videoconferencing psychotherapy for addiction are still embryonic. Despite the many advantages already mentioned, measurements of the observer to evaluate the alliance in videoconferencing psychotherapy are rare. The results here indicate that the Brazilian version of the WAI-SR-O can be a reliable measurement for the working alliance in videoconferencing psychotherapy for alcohol addiction. However, attention needs to be given to training, especially for the task area and observers with differing clinical experience. The weak correlations among the WAI-SR-O, WAI-T, and WAI-C indicate that we have not corroborated the relationship with external variables, although these have been discussed as several limitations of the instruments, such as lack of validation for Brazil. However, the results showed that observers, clients, and therapists evaluate an alliance in different ways. Similar results have been found in other studies, and this needs to be better investigated for videoconferencing psychotherapy in future studies.

Considering prospects for increasing online psychotherapy practice, the WAI-SR-O can contribute to research and clinical training programs. The availability of valuable tools for the study on videoconferencing psychotherapy is essential for a better understanding of its functioning and to improve assistance in this modality.

## Data Availability Statement

The raw data supporting the conclusions of this article will be made available by the authors, without undue reservation.

## Ethics Statement

The studies involving human participants were reviewed and approved by Research Ethics Committee of Federal University of Juiz de Fora (protocol number 1.360.973). The patients/participants provided their written informed consent to participate in this study.

## Author Contributions

NR and LS: writing—original draft. NR, FC, NK, and LS: writing—review and editing and cross-cultural adaptation. NR and FC: statistical analysis. NR, FC, and LS: interpretation of statistical analysis. All the authors contributed to the article and approved the version submitted.

## Conflict of Interest

The authors declare that the research was conducted in the absence of any commercial or financial relationships that could be construed as a potential conflict of interest.

## Publisher's Note

All claims expressed in this article are solely those of the authors and do not necessarily represent those of their affiliated organizations, or those of the publisher, the editors and the reviewers. Any product that may be evaluated in this article, or claim that may be made by its manufacturer, is not guaranteed or endorsed by the publisher.
